# How “Greasers” Live

**Published:** 1883-06

**Authors:** 


### HOW "GREASERS" LIVE.

The word "Greaser" is applied to the common native or Indian Mexican. There is a great and marked difference, like a caste distinction, between the "Greaser" Mexican and the Spanish Mexican, who boasts of a decent from pure Castilian blood. The former is lazy, thriftless, ignorant, superstitious and unstable. The latter is usually bright, active and intelligent. As may be expected, they, in harmony with the white element, control most of the wealth and commerce of the country. In the Northern States of Old Mexico the greatest portion of the people are the former class, and hence the governments are unstable and subject to frequent revolution under the influence of factious leaders. In the more southerly States there is a greater diffusion of the Castilian blood, and revolutions are more rare and difficult. On the ranch and village home of the "Greaser" Mexican eveiything bears the stamp of negligence and shiftlessness. Their gaunt, sharp-nosed, long-legged and tan-colored hogs share with their owners in the comforts of the family residence. No fences except brush surround their fields. Generally there are none. They raise just sufficient wheat, barley, beans and chili (red peppers) to meet their absolute needs. They thrash their crops upon bare, smooth ground by driving flocks of goats over them and washing in the nearest stream. They often plough with a crooked stick, and the hoe is their scythe sickle, and reaper. Even their hay is cut with a hoe. They as a rule live in villages and cultivate small fields upon their outskirts. Living as they do, and possessing a soil which under irrigation is wonderfully productive, they require but little ground to cultivate. For this reason at points between their villages there are large tracts of unoccupied lands subject to entry, which are being rapidly occupied by Americans.

An impression prevails somewhat at the North and East that the inertia of this people is due largely to some enervating influence of the climate. This is an error. The climate is superb, and the air exhilarating rather than enervating. I have been here in all seasons, and know of no climate where I can exert myself so freely with so little exhaustion. This is the universal record of American settlers. Rarely does the mercury range over ninety degrees in the hottest summer-day, and the summer heats are almost invariably tempered by pleasant breezes. I have never heard of a case of sunstroke here. The nights are always cool, requiring blankets for comfort, and free from mosquitoes, and sleep is sweet and restful. I have never seen a mosquito-bar in New Mexico, nor have I ever felt the need of one. As I have said before, taken as a whole the country is not beautiful nor attractive to the eye. The mountain scenery is at many points wonderfully beautiful and picturesque. But apart from the mountains and the few river valleys the country is one vast cattle range. —*Correspondence of the Chicago Tribune.*

